# An Adult Patient with Systemic Mastocytosis and B-Acute Lymphoblastic Leukemia

**DOI:** 10.1155/2014/526129

**Published:** 2014-11-06

**Authors:** Theodoros Iliakis, Niki Rougkala, Panagiotis T. Diamantopoulos, Vasiliki Papadopoulou, Fani Kalala, Konstantinos Zervakis, Nefeli Giannakopoulou, Polixeni Chatzinikolaou, Georgia Levidou, Eleftheria Lakiotaki, Penelope Korkolopoulou, Efstratios Patsouris, Eleni Variami, Nora-Athina Viniou

**Affiliations:** ^1^Hematology Unit, First Department of Internal Medicine, Laikon General Hospital, National and Kapodistrian University of Athens, 17 Agiou Thoma Street, 11527 Athens, Greece; ^2^Department of Pathology, National and Kapodistrian University of Athens, Athens, Greece

## Abstract

Mastocytosis is a myeloproliferative neoplasm characterized by clonal expansion of abnormal mast cells, ranging from the cutaneous forms of the disease to mast cell leukemia. In a significant proportion of patients, systemic mastocytosis (SM) coexists with another hematologic malignancy, termed systemic mastocytosis with an associated hematologic nonmast cell lineage disorder (SM-AHNMD). Despite the pronounced predominance of concomitant myeloid neoplasms, the much more unusual coexistence of lymphoproliferative diseases has also been reported. Imatinib mesylate (IM) has a role in the treatment of SM in the absence of the KITD816V mutation. In the setting of SM-AHNMD, eradicating the nonmast cell malignant clone greatly affects prognosis. We report a case of an adult patient with SM associated with B-lineage acute lymphoblastic leukemia (B-ALL). Three cases of concurrent adult ALL and mastocytosis have been reported in the literature, one concerning SM and two concerning cutaneous mastocytosis (CM), as well as six cases of concomitant CM and ALL in children.

## 1. Introduction

Mastocytosis is a clonal myeloproliferative disorder characterized by dysregulation of various organs infiltrated with abnormal mast cells and by symptoms attributed to histamine release. According to the location of mast cell proliferation it is classified into cutaneous mastocytosis (CM), affecting solely the skin, and to systemic mastocytosis (SM), involving at least one extracutaneous organ with more severe clinical manifestations. SM is further divided to six subtypes, according to the recent World Health Organization (WHO) classification, reflecting progressive mast cell clonal expansion and severity of symptoms [1].

In up to 40% of cases, SM is accompanied by a nonmast cell hematologic disorder (SM-AHNMD) [1], resulting in a combination of symptoms, related to each separate component [2]. A form of reactive mast cell hyperplasia, which is observed in other hematologic neoplasms, such as lymphoplasmacytic lymphoma and hairy cell leukemia (HCL) must be excluded [3]. A predominance of associated myeloid disorders, especially chronic myelomonocytic leukemia (CMML) is reported in SM-AHNMD [4, 5]. Lymphoproliferative neoplasms are much less commonly implicated [4, 5], referring to 10 reported cases of non-Hodgkin lymphomas (NHL), 3 cases of chronic lymphocytic leukemia/small lymphocytic lymphoma (CLL/SLL), 1 case of HCL, 6 cases of multiple myeloma (MM), and 1 case of Hodgkin lymphoma (HL). SM associated with adult acute lymphoblastic leukemia (ALL) has been documented in the case of a patient with SM associated with B-ALL carrying the (13;13) (q12;q22) translocation [6]. Two other cases concerning adults with concurrent CM and ALL have also been reported [7, 8]. Among children with ALL, six cases of concomitant CM have been described [8–11].

Identification of the KITD816V mutation comprising almost 80% of c-kit mutations [5] is of major importance in SM-AHNMD. Cases of SM with wild type c-kit or those who carry c-kit mutations other than D816V may respond to therapy with imatinib mesylate (IM). D816V mutation, with rare exceptions, confers resistance to tyrosine kinase inhibitors (TKI) [12]. We present the case of a young woman with B-ALL and concurrent SM lacking the KITD816V mutation.

## 2. Case Report

**Figure 1:**
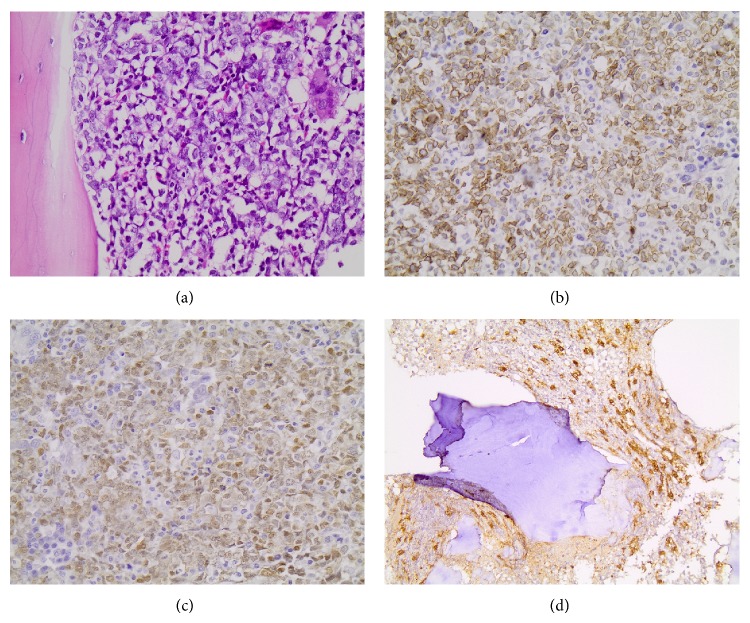
Initial bone marrow trephine biopsy showing infiltration by an immature blastoid population in H&E stain (a), which immunohistochemically was positive for CD79a (b) and TDT (c). The presence of a small aggregate consisting of spindle shaped mast cells is also illustrated (d).

A 40-year-old Caucasian female was admitted displaying symptoms of weakness and fatigue, being febrile (37.9°C) with moderate pallor. Her liver was palpable, as well as a slightly enlarged left inguinal lymph node. She also manifested diffuse cutaneous brown macular lesions on her trunk. Her complete blood count (CBC) revealed normocytic normochromic anemia with a normal leukocyte count and moderate thrombocytopenia. Bone marrow (BM) trephine biopsy and immunophenotype showed extensive infiltration from B-ALL expressing the surface markers CD10, CD19, CD22, CD79a, CD34, CD123, CD38, and Tdt, with an aberrant coexpression of the myeloid markers CD13, CD33. Eosinophilia was noted and spindle-shaped mast cells were present, scattered or in small aggregates, being positive in c-kit and negative in CD2 staining (Figure 1). Polymerase chain reaction (PCR) for KITD816V mutation, fibroblast growth factor receptor 1 and platelet derived growth factor receptor (FGFR1, PDGFR) rearrangements, and breakpoint cluster region/Abelson tyrosine kinase (BCR/ABL) fusion gene was negative. Conventional cytogenetics was normal in all studied metaphases.

**Figure 2:**
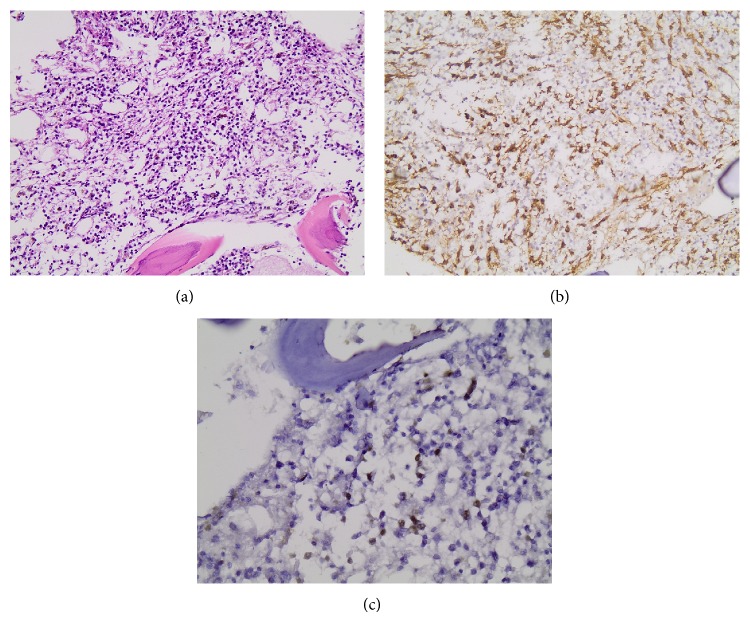
Bone marrow trephine biopsy after induction with an extensive infiltration by spindle shaped mast cells as illustrated in H&E stain (a) as well as c-KIT immunostaining (b). Immunohistochemical expression for TDT also revealed the presence of infiltration by TDT (+) lymphoblasts (c), which however was lower than that observed initially.

The patient received induction therapy for B-ALL, consisting of dexamethasone, vincristine, idarubicin, cyclophosphamide, cytarabine, and thioguanine, along with intrathecal methotrexate. During induction, she developed severe low respiratory tract infections. BM immunophenotyping and trephine biopsy following induction revealed residual leukemic disease consisting of 10% lymphoblasts and an extensive mast cell infiltration exceeding 50% of nucleated BM cells (Figure 2). The majority of mast cells (>25%) were spindle-shaped, distributed either in a diffuse pattern or forming dense aggregates of more than 15 cells, synchronously expressing the surface markers c-kit (CD117) and CD2. Serum tryptase levels were normal. Thus, the major criterion and two out of the four minor recent diagnostic WHO criteria for SM were fulfilled, unequivocally establishing the diagnosis of SM-AHNMD, in terms of the preexisting B-lymphoid neoplasm [1]. A subsequent skin biopsy revealed only sparse mast cells in the dermis.

IM was administered orally to our patient in a daily dose of 100 mg, due to its previously outlined effectiveness in wild type c-kit and other than D816V mutated cases of SM [12]. Hematological response was temporarily obtained, with CBC values reaching near normal levels in 8 days but it had short duration (Table 1). Since our patient failed to achieve ALL complete remission (CR), she received a salvage regimen consisting of idarubicin, fludarabine, and cytarabine.

**Figure 3:**
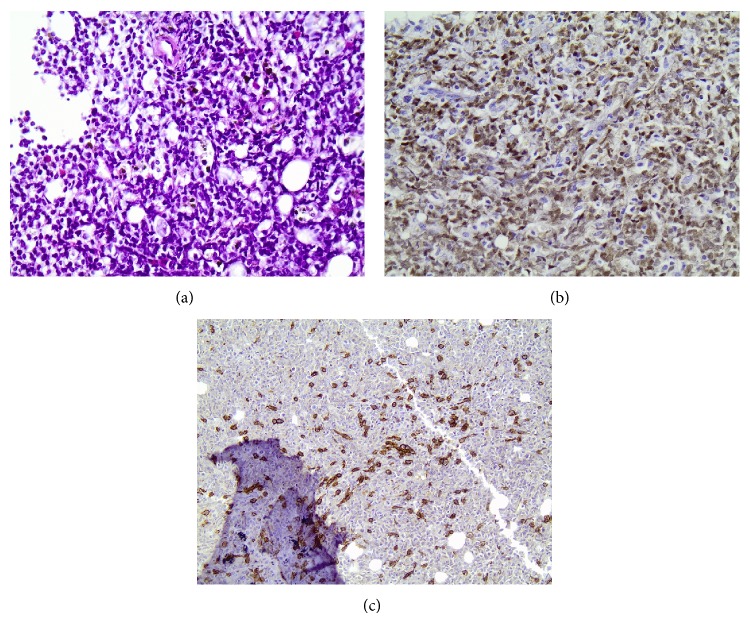
Final bone marrow trephine biopsy in which there is extensive infiltration by medium sized lymphoblasts (a), which were immunohistochemically positive for TDT (b). Concurrent mast cell infiltration is also observed (c-KIT immunohistochemical analysis (c)).

Following two cycles of the salvage regimen, the patient experienced disease progression. Infusion of clofarabine, a purine nucleoside antimetabolite, was ineffective. IM administration was interrupted, because of elevated liver enzymes. Subsequent BM immunophenotyping and trephine biopsy indicated extensive lymphoblastic marrow infiltration up to fifty percent, with concurrent mast cell infiltration (Figure 3). Palliative care was administered to the patient, who died after a severe low respiratory tract infection.

## 3. Discussion

SM-AHNMD is a distinct form of SM characterized by synchronous evolution of two separate clonal populations, one consisting of mast cells and one as a second hematologic malignancy. CMML is the most prevalent concomitant hematologic disorder, followed by other myelodysplastic/myeloproliferative syndromes (MPN/MPDs), MPNs, MDS, and acute myeloid leukemia (AML) [4, 5]. Lymphoproliferative neoplasms are much less frequently found in this setting with reported cases of CLL/SLL, HCL, plasma cell dyscrasias, and lymphomas [3–5]. Among the latter, HL, splenic marginal zone lymphoma, diffuse large B-cell lymphoma, hepatosplenic T-cell *γ*/*δ* lymphoma, and cutaneous B-cell lymphoma have rarely been described. We are currently aware of a sole published case of an adult with KITD816V mutated SM associated with B-ALL  carrying  a (13;13) (q12;q22) translocation [6]. The authors reported that B-ALL was cured by alloHSCT, while SM persisted for more than a decade. The other two reported adult patients suffered from concomitant ALL with cutaneous forms of mastocytosis [7, 8].

SM-AHNMD constitutes about 30%–40% of all SM cases [4, 5] and harbors certain difficulties in terms of diagnosis, therapeutic management, and response assessment. The diagnosis of occult SM associated with ALL is highly demanding, due to its rarity along with shared clinical manifestations between the two entities. In our patient, initial BM biopsy revealed only a 15% infiltration of mast cells, whereas constitutional symptoms and hepatomegaly were attributed to the lymphoid neoplasm.

The combined nature of SM-AHNMD underlies the need to confront each malignant entity separately but simultaneously. Both its clinical course and prognosis strongly correlate with those of the associated nonmast cell neoplasm and standard regimens are required for the latter. Management of histamine related symptoms, such as anaphylaxis, pruritus, flashing, or malabsorption, lies on relevant pharmaceutical agents. Interferon-*α*, corticosteroids, and cytoreductive therapies, mostly cladribine, have been used to treat the SM component, with minor responses [13]. Data on the beneficial role of allo-HSCT are scarce and conflicting, due to the rarity of relevant cases [14]. In addition, leukemias arising in the context of SM are considered of poor risk [13]. This was also the case for our patient, who was primarily refractory to all treatment modalities.

IM has demonstrated effectiveness only in c-kit wild type SM cases or when mutations in c-kit outside exon 17 are present [12]. In contrast to CMML cases and other myeloid neoplasms which usually exhibit KITD816V positivity that is not a prominent feature in the category of concomitant lymphoid neoplasms with SM [5], our patient, who did not carry the KITD816V mutation, was treated with IM.

The duration of response is a prerequisite for its estimation according to the recent International Working Group-Myeloproliferative Neoplasms Research and Treatment (IWG-MRT) & European Competence Network on Mastocytosis (ECNM) consensus criteria, taking into account ascites and pleural effusions, spleen size, liver function, serum tryptase, albumin and CBC values, and mast cell infiltrates in tissue biopsy sections [15]. Prior to duration of response, assessing organ damage is the most crucial parameter in order to apply the previously mentioned criteria in SM-AHNMD cases and may prove difficult due to shared clinicopathological features between the two components of the disease [15]. In our case, response to IM administration could not be assessed because of chemotherapy-related toxicity. Eligible organ damage mostly consisted of transfusion dependent anemia and thrombocytopenia, due to BM infiltration from both diseases. Infiltration from the ALL clone in the performed BM biopsy was around 10% and, thus, not compatible with the severity and duration of cytopenias. Liver biopsy was not carried out due to the patient's poor performance status. As anticipated, the clinical course and prognosis of our patient were principally determined by her poor response to all applied antileukemic treatment modalities, failing to achieve CR. We should acknowledge that the concurrent SM complicated the chemotherapy related marrow and liver toxicities. IM administration enhanced transient hematologic improvement and permitted the administration of subsequent chemotherapeutic agents for ALL. In conclusion, the diagnosis of SM-AHNMD requires careful consideration of both clinical and laboratory findings. The choice of treatment depends on the nature and clinical behaviour of both disorders [13].

Off-label drug use: clofarabine for acute lymphoblastic leukemia in adults.

## Figures and Tables

**Table 1:** Hematological response of our patient to imatinib mesylate (IM).

	Day of assessment of response following induction (concurrent G-CSF usage)	Initiation of IM 6 days later (cessation of G-CSF)	5 days following introduction of IM (without G-CSF or transfusional support)	8 days following introduction of IM (without G-CSF or transfusional support)	15 days following introduction of IM (initiation of salvage therapy)
Hemoglobin (gr/dL)	7.2	8.6	11.7	12.3	11.2
Neutrophils (/*μ*L)	0.0	1100	3600	4180	2900
Platelets (/*μ*L)	11000	16000	91000	180000	170000

G-CSF; granulocyte-colony stimulating factor.
